# Comparison of work efficiency in factory workers: pre & post covid lockdown – a cross sectional study

**DOI:** 10.1186/s12889-023-15886-3

**Published:** 2023-05-24

**Authors:** Prathamesh Kotagi, Mubashir Angolkar, Rajashree Koppad

**Affiliations:** Department of Public Health, J. N. Medical College, KLE Academy of Higher Education & Research, Belgavi, Karnatka India

**Keywords:** Work efficiency, Factory workers, COVID lockdown, Pre-lockdown, Post-lockdown, Employee performance, Industrial sector, Pandemic impact, Workplace interventions

## Abstract

**Background:**

The COVID-19 pandemic's impact on economies worldwide has caused changes in work patterns, reduced productivity, and job losses, particularly affecting factory workers. Lockdown measures have also led to reduced physical activity levels, which is a significant risk factor for chronic diseases. This study aims to investigate efficiency of factory workers pre and post lockdown periods. The findings will contribute to identifying evidence-based strategies to minimize the negative impact of lockdown measures on factory workers' productivity and health.

**Materials and methods:**

A cross-sectional study was conducted to assess the work performance of employees in a medicine manufacturing unit. Data was collected from factory workers online and study period was January 2021 to April 2022. Survey includes close ended questions regarding work performance of employees before lockdown period (Before 20/03/2020) and performance after lockdown period (After August 2020). The sample size of 196 employees selected through simple random sampling. A questionnaire consisting of demographic factors, work details, and work performance was prepared using pretested standard tools, including the Individual Work Performance Questionnaire (IWPQ), the World Health Organization Health and Work Performance Questionnaire (HPQ), and the Stanford Presenteeism Scale (SPS-6). The collected data was analysed using descriptive statistics and a paired t-test.

**Results:**

The study found that prior to lockdown, 99% of employees consistently had higher performance, with 71.4% ranking in the top 10. However, after lockdown, the percentage of employees with high performance decreased to 91.8%, with only 63.3% ranking in the top 10. These differences were statistically significant, indicating a decrease in work efficiency of 8.1%. Before lockdown, employees worked longer hours, including on off days, while after lockdown, a small proportion missed work due to various reasons, resulting in better quality work.

**Conclusion:**

In conclusion, the study highlights the significant impact of the COVID-19 pandemic on the work efficiency of factory workers. The findings indicate a decrease in work efficiency after the lockdown, coupled with an increase in employee stress. This suggests that the pandemic has brought about unique challenges for factory workers that need to be addressed to maintain their well-being and productivity. This study emphasizes the importance of creating a supportive work environment that prioritizes the mental and physical health of employees, especially during times of crisis.

## Background

The COVID-19 epidemic impacted every country on the planet. The global devastation and despair caused by the pandemic are unprecedented in recent history. The pandemic has triggered a severe economic catastrophe, sending many countries into recession. As per the International Monetary Fund (IMF), the global financial system has negotiated by 4.9 percent in 2020. The pandemic and national lockdown disturbed the lives of employees working in the private sector. There was limited discussion in the news outlets about the challenges facing workers in manufacturing jobs in the private sector [1].

The World Health Organization (WHO) has issued advice on how to stop the spread of COVID 19 at work on a worldwide platform. When arranging meetings, activities, and getting the organization ready, the guidelines comprise COVID 19 risk management measures. As employees are forced to either remain at home or work with a minimal team, such guidelines have the overall consequence of either decreasing or ceasing production. Due to the coronavirus pandemic, predictions for annual worldwide Gross domestic product (GDP) raise for 2020 have declined point to 2.4 percent, according to the Organization for Economic Co-operation and Development (OECD) Economic Outlook Interim Report of March 2020. The coronavirus pandemic, on the other hand, might limit world growth to 1.5 percent. COVID 19 is a major threat to the millions of small and medium enterprises (SMEs) around the globe, especially in the most dependent nations. SME's employ more than 90 percent of the people in developing nations, yet they confront limits on exports, restricted borders, declining demand, and a dramatic slowing of worldwide supply chains [2, 3].

The economic impact of the epidemic and response actions such as the Government of India's national lockdown in March was felt acutely in India, with the country's Gross Domestic Product (GDP) dropping by a stunning 23.9 percent in the first quarter of the fiscal year 2020–2021 [1, 4–7].

Coronavirus outbreak were pressed almost all of the workers worldwide to operate in a very different environment compared to what it was previously. Coronavirus outbreak social separation, restrictions on travel, online or distant work, and minimal workers are all factors to consider are all examples of triggered interventions that affect the continuation of earlier method, leading change in work. Actions during the staff behaviour changed as a result of the Coronavirus outbreak. Managers and supervisors, group leaders, and experts are all in this category. Concerned about such lifestyle modifications since they can have an impact on employees' psychological, intellectual, and physiological well-being, affecting deliveries and productivity [8–10].

Coronavirus outbreak prompted enterprises to take action to mitigate its influence on efficiency. The effect in ambiguous way, as there are cause for low and positive efficiency [11].

Coronavirus outbreak and other life's traumatic events can have a serious affect on a personal cognitive efficiency and mental wellbeing. Tension, anxiety, mental disorientation, social deprivation, and anxiety are just a few of the cognitive disorders that accompany personal confined experiences as a result of COVID-19, which resulted in stress, dread, and dissatisfaction. Employee performance (EP) has been shown to be affected by work stress in unpredictable situations such as the onset of the COVID-19 pandemic [12].

Organizations are constantly struggling to survive. Uncertain conditions, like as the Coronavirus outbreak, might cause workers to they feel anxious, and their performance suffers as a result. Workers' mind drifts from their job by the threats that arise in the work setting as an outcome of outbreaks, which endanger their safety at work by making health at sick. Personnel could be stressed due to change which could lead to conflicts that interrupt people's workflow and contribute to poor efficiency [13–15].

The present study was planned to assess work patterns and work efficiency among factory workers during the lockdown. The repercussions of the change on efficiency and the length of period they spent in work-place.

A descriptive study was done in 2020 at an industrial area, South Tangerang, Indonesia. The study included 96 participants. This study showed that COVID-19 pandemic effect on productivity impacted by a layoff in an industrial area. In that study, they found that many sectors got into trouble, especially the industrial sector, due to the pandemic situation. Manufacturing units suffered more. The pandemic effect has a negative influence on productivity. Also, they found that productivity negatively influences workers' layoffs. Termination of workers was not taken into consideration in regards to productivity. Pandemic situations and productivity both are independent, but they may not apply to all areas of the industrial sector [16–18].

A descriptive case study carried out at Masvingo, Zimbabwe in 2020, showed that the small to medium industry sector was heavily damaged by the Coronavirus outbreak and the subsequent shutdown enacted to stop the virus transmission resulted in the shut off all industries in Masvingo. This leads to the significant decline in manufacturing, necessitating the layoff of temporary employees and attachés. COVID-19 had a net effect on industries, lowering productivity dramatically. For that reason, the mandate to limit people's movement directly impacted their productivity [2, 19].

The methods used in this research are appropriate for studying the impact of the COVID-19 pandemic on factory workers' work efficiency. The study used a cross sectional research design that included a survey questionnaire as the data collection tool. The use of a survey questionnaire is an appropriate method for collecting data from a large number of respondents, which is necessary to generalize the findings to the entire population of factory workers. Additionally, the survey questionnaire allowed for the collection of data on a wide range of variables, including work efficiency, stress levels, and work conditions, which were critical to understanding the impact of the pandemic on the workers' productivity.

The survey questionnaire used in the study included both closed-ended and Likert-scale questions. The use of closed-ended questions allowed for the collection of standardized data, which facilitated data analysis and interpretation. Furthermore, the use of Likert-scale questions allowed for the measurement of respondents' attitudes, perceptions, and opinions, which provided valuable insights into how the workers were coping with the pandemic-induced changes in their work environment.

The research methods used in the article provide valuable insights into the impact of the COVID-19 pandemic on factory workers' work efficiency. The findings of the study highlight the need for employers to take into account the unique challenges faced by employees during pandemics and to implement measures to support their well-being and maintain their work efficiency.

The primary objective of this study is to analyze the work efficiency of factory workers before and after the lockdown. Various parameters such as employee punctuality, performance, quantity, and quality of work will be evaluated to understand the impact of the pandemic on work efficiency. The study will also assess the level of stress experienced by factory workers due to changes in the work environment brought about by the pandemic.

The secondary objective of this study is to understand the work pattern of factory workers and explore the association between the nature of work and work efficiency. By studying the work pattern, it will be possible to identify areas that may require improvements to enhance work efficiency.

Research is needed to investigate the impact of COVID-19 induced lockdown measures on the work efficiency or productivity of factory workers and to find out possible reasons behind it and measures for improving work efficiency or productivity during such lockdown periods. By conducting this study, we can identify evidence-based strategies that can help minimize the negative impact of lockdown measures on the productivity and health of factory workers and hence fulfilment of research question “How much work efficiency of factory workers is affected due to lockdown?”.

## Materials and methods

The study aimed to assess the work performance of employees working in a medicine manufacturing unit. A cross-sectional study design was employed, and study period is from January 2021 to April 2022. The study population consisted of all workers employed in the factory, and the sample size was calculated using the formula *n* = Z^2^Npq / (N-1) d^2^ + Z^2^pq (where, Z-value = 1.96, *p* = Taking anticipated prevalence 50% = 0.5, q = 1—p = 1 – 0.5 = 0.5, *N* = Known population, D = Allowable error = 10%), with a sample size of 196, formula used for calculating sample size is commonly known as the formula for calculating sample size for estimating a population proportion using a confidence interval. It is also sometimes referred to as the margin of error formula. The inclusion criteria for the study were all employees working in the company, while the exclusion criterion was employees who were not willing to participate in the research. A simple random sampling method was used to select the participants, and computer-generated randomization was carried out.

The data collection process involved obtaining online informed consent from the participants, and an online web-based Google form was prepared and circulated through email. Questionnaire includes close ended questions regarding work performance of employees before lockdown period (Before 20/03/2020) and after lockdown (After August 2020). The questionnaire consisted of information on demographic factors, details on the nature of work, and work performance, using three pretested and predesigned standard tools, including the Individual Work Performance Questionnaire (IWPQ) [20, 21], the World Health Organization Health and Work Performance Questionnaire (HPQ) [22], and the Stanford Presenteeism Scale (SPS-6) [23]. The IWPQ assessed key job effectiveness characteristics, using a 5-point rating scale, while the HPQ was a self-report questionnaire that estimated medical issues in the work-setting with respect to impaired employee productivity, sick leave, and work-related injuries. The SPS-6 assessed the influence of health conditions on work engagement and total perceived output, using a five-point Likert scale.

The collected data was entered into M.S. Excel-19 and analyzed using SPSS version-20. Descriptive statistics were used to analyze the demographic profile and work performance variables. A paired t-test was used to identify any significant difference between pre-lockdown and post-lockdown work efficiency. In short, the study assessed the work performance of employees working in factory, and three pretested and predesigned standard tools were employed to collect data. The collected data was analyzed using descriptive statistics and a paired t-test.

Ethical clearance approval for this study was obtained from the JNMC Institutional Ethics Committee on Human Subject and Research, J.N. Medical College, Belagavi, Karnataka, India. The research described in this article was conducted in accordance with the ethical standards set forth by the JNMC Institutional Ethics Committee on Human Subject and Research, J.N. Medical College, Belagavi, Karnataka, India. All data were collected and analyzed in accordance with relevant ethical guidelines and regulations.

### Result

The present study aimed to investigate various aspects of employees' performance and well-being before and after the lockdown. The findings revealed that the majority of employees belonged to the age group of 41–45 years (24.5%), and the male-to-female ratio of factory workers was 76% to 24%. Most employees had 0–10 years of service (53.6%), and a significant proportion belonged to the laborer category (44.4%). Before the lockdown, employees worked long hours, came in early or stayed late, and worked on off days. After the lockdown, 94.4% of employees continued to work in the same manner, with only a small proportion missing work due to vacation, physical or mental health problems, or other reasons.

The Performance scale as showed in Table 1 depicted the distribution of employees according to their performance ranks. Before the lockdown, the majority of employees (71.4%) were top performers, while a small proportion belonged to rank 7 (1.5%). After the lockdown, the number of top performers decreased (63.3%), while the number of employees on rank 7 increased (8.2%). However, the number of employees on ranks 8 and 9 remained almost the same. The paired T test results as mentioned in Table 2 indicated that the difference between work performance before and after the lockdown was statistically significant (*p* value: 0.00001; *p* value < 0.05), signifying the impact of the lockdown on employee performance.

**Table 1:** Distribution of employees according to scale of performance

Scale of performance	Before Lockdown		After Lockdown	
	**Frequency**	**Percent**	**Frequency**	**Percent**
7	3	1.5	16	8.2
8	26	13.3	28	14.3
9	27	13.8	28	14.3
10—Top Performance	140	71.4	124	63.3
Total	196	100.0	196	100.0

**Table 2:** Paired T test result of work performance before and after lockdown

	Paired Differences					t	df	Sig. (2-tailed)
	Mean	Std. Deviation	Std. Error Mean	95% Confidence Interval of the Difference				
				Lower	Upper			
Work Performance before lockdown—Work Performance after lockdown	0.22449	0.73073	0.05219	0.12155	0.32743	4.301	195	0.00001

Regarding the quantity and quality of work as mentioned in Tables 3 and 4, the majority of employees had very good quantity (95.9%) and much better quality (95.4%) of work before the lockdown. After the lockdown, the number of employees with very good quantity of work decreased slightly (87.2%), while the number of employees with good quantity of work increased (12.2%). However, the number of employees with much better quality of work decreased slightly (86.7%), while the number of employees with better quality of work increased (7.1%). Only a small proportion of employees had good quality of work after the lockdown.

**Table 3:** Distribution of employees according to quantity of work

Quantity of work	Before Lockdown		After Lockdown	
	**Frequency**	**Percent**	**Frequency**	**Percent**
Somewhat Sufficient	-	-	1	0.5
Good	8	4.1	24	12.2
Very Good	188	95.9	171	87.2
Total	196	100	196	100

**Table 4:** Distribution of employees according to quality of work

Quality of work	Before Lockdown		After Lockdown	
	**Frequency**	**Percent**	**Frequency**	**Percent**
Better	9	4.6	14	7.1
Good	-	-	12	6.1
Much Better	187	95.4	170	86.7
Total	196	100	196	100

Furthermore, the study examined the distribution of employees' mental health assessment related to stress, ability to finish work, experiencing pleasure in work, feeling hopeless to finish tasks, ability to achieve goals despite health problems, and feeling energetic at work before and after the lockdown; as showed in Tables 5, 6, 7, 8, 9 and 10. The results showed that the majority of employees strongly disagreed that due to health problems, the stress of work was hard to handle before the lockdown (99.5%). However, after the lockdown, four (2%) employees agreed to the same statement, while only one (0.5%) employee disagreed. A small proportion of employees remained neutral, indicating the impact of the lockdown on employees' mental health.

**Table 5:** Distribution of employees according to mental health assessment regarding stress

Due to health problem stress of work much hard to handle	Before Lockdown		After Lockdown	
	**Frequency**	**Percent**	**Frequency**	**Percent**
Agreed	-	-	4	2.0
Disagreed	1	0.5	1	0.5
Neutral	-	-	6	3.1
Strongly Disagreed	195	99.5	185	94.4
Total	196	100	196	100

**Table 6:** Distribution of employees regarding ability to finish work

Despite of health problem able to finish work	Before Lockdown		After Lockdown	
	**Frequency**	**Percent**	**Frequency**	**Percent**
Agreed	91	46.4	93	47.4
Strongly Agreed	104	53.1	99	50.5
Disagreed	-	-	1	.5
Neutral	-	-	3	1.5
Strongly Disagreed	1	.5	1	.5
Total	196	100	196	100

**Table 7:** Distribution of mental health assessment regarding distraction in work

Health problem distracted to take pleasure in work	Before Lockdown		After Lockdown	
	**Frequency**	**Percent**	**Frequency**	**Percent**
Agreed	1	0.5	4	2.0
Strongly Agreed	-	-	-	-
Disagreed	1	0.5	2	1.0
Neutral	-	-	4	2.0
Strongly Disagreed	194	99.0	186	94.9
Total	196	100	196	100

**Table 8:** Distribution of employees regarding despair to finish work

Due to health problem feeling hopeless to finish task	Before Lockdown		After Lockdown	
	**Frequency**	**Percent**	**Frequency**	**Percent**
Agreed	-	-	-	-
Strongly Agreed	-	-	-	-
Disagreed	1	0.5	4	2.0
Neutral	-	-	-	-
Strongly Disagreed	195	99.5	192	98.0
Total	196	100	196	100

**Table 9:** Distribution of employees regarding ability to achieve goal

Despite health problems can achieve goals	Before Lockdown		After Lockdown	
	**Frequency**	**Percent**	**Frequency**	**Percent**
Agreed	87	44.4	86	43.9
Strongly Agreed	109	55.6	108	55.1
Disagreed	-	-	1	0.5
Neutral	-	-	1	0.5
Strongly Disagreed	-	-	-	-
Total	196	100	196	100

**Table 10:** Distribution of employees regarding feeling energetic in work

Felt energetic to complete work	Before Lockdown		After Lockdown	
	**Frequency**	**Percent**	**Frequency**	**Percent**
Agreed	84	42.9	85	43.4
Strongly Agreed	112	57.1	109	55.6
Disagreed	-	-	-	-
Neutral	-	-	2	1.0
Strongly Disagreed	-	-	-	-
Total	196	100	196	100

The present study investigated the impact of lockdown on employees' ability to handle work-related stress, as well as their feelings of despair and pleasure at work. Before the lockdown, 91 (46.4%) employees agreed that they were able to finish their work despite their health problems, while 104 (53.1%) employees strongly disagreed with the statement. However, after the lockdown, 93 (47.4%) employees agreed to the same statement, 3 (1.5%) employees remained neutral, 99 (50.5%) employees strongly agreed, and only one employee strongly disagreed.

Regarding experiencing pleasure in work, only one employee (0.5%) agreed that health problems distracted them from experiencing pleasure in work before the lockdown, while 194 (99%) employees strongly disagreed. After the lockdown, four (2%) employees agreed that health problems distracted them from experiencing pleasure in work, two (1%) employees disagreed, and four (2%) employees remained neutral. Additionally, 186 (94.9%) employees strongly disagreed that health problems distracted them from pleasure in work.

The Table Distribution of Employees Regarding Despair to Finish Work indicated that before the lockdown, only one employee (0.5%) disagreed that due to health problems, they felt hopeless to finish their tasks, while 195 (99.5%) employees strongly disagreed. However, after the lockdown, four (2%) employees disagreed with the statement, while 192 (98%) employees strongly disagreed.

Table Distribution of Employees Regarding Ability to Achieve Goal revealed that before the lockdown, 87 (44.4%) employees agreed that despite health problems, they can achieve their goals, and 109 (55.6%) employees strongly agreed. After the lockdown, 86 (43.9%) employees agreed, and 108 (55.1%) employees strongly agreed. One (0.5%) employee disagreed, and one (0.5%) employee remained neutral to the statement.

With respect to feeling energetic at work, before the lockdown, 84 (43.9%) employees agreed that they felt energetic to complete their work, and 112 (57.1%) employees strongly agreed. After the lockdown, 85 (43.4%) employees agreed, and 109 (55.6%) employees strongly agreed. Two (1%) employees remained neutral to the statement.

Finally, all employees (100%) understood the questionnaire and found it relatable to their work. These findings demonstrate the significant impact of lockdown on employees' mental health, particularly in terms of their ability to manage work-related stress and despair to finish their tasks. However, the majority of employees expressed confidence in their ability to achieve their goals and feel energetic at work, despite their health problems. These results highlight the need for interventions to support employees' mental health and well-being in the workplace.

## Discussion

The COVID-19 pandemic has caused unprecedented disruptions in every aspect of our lives, including the global economy and workforce, with factory workers being severely affected by pandemic-induced lockdowns [1]. This study aimed to investigate the impact of lockdowns on the work efficiency of factory workers. The results showed that work efficiency was significantly affected by the lockdown, with a decrease in work performance observed after the implementation of the lockdown. Before the lockdown, the factory workers had a normal work routine, which was disrupted by the sudden lockdown. The workers were forced to work from home or take a break from work, resulting in a significant decline in work efficiency. The study found that the workers faced several challenges while working from home, including a lack of proper infrastructure and equipment, distractions, and difficulty in maintaining communication with colleagues. The COVID-19 pandemic and subsequent lockdowns have had a profound impact on businesses and industries worldwide, leading to widespread lockdowns in many countries. This study aimed to investigate the work efficiency parameters of factory workers before and after lockdown and to determine the association between the nature of the work and work efficiency. The results of the study indicate that the lockdown has had a significant impact on the work efficiency of factory workers.

Our findings revealed that the lockdowns had a significant negative impact on the work efficiency of factory workers. The workers reported a decrease in work productivity, increased fatigue and stress levels, and difficulty in maintaining work-life balance [2, 11]. The lockdown-induced isolation and social distancing measures have also affected the workers' mental health and well-being, leading to absenteeism and decreased work motivation [24, 25]. Moreover, the nature of work was found to have a significant association with work efficiency. The workers engaged in physically demanding jobs, such as assembly line work, reported a more significant decline in work efficiency than those with desk jobs. [16, 26]. The lack of physical activity and the sedentary nature of desk jobs have also been linked to decreased work efficiency and mental health issues [27–29]. To mitigate the negative effects of lockdowns on work efficiency, several strategies can be implemented. Providing mental health support and promoting physical activity and exercise can help workers cope with the stress and isolation induced by lockdowns [30, 31]. Flexible work arrangements, such as remote work and flexible working hours, can also help workers achieve a better work-life balance [32–34].

The majority of the employees were in the age group of 41 to 46 years, and the mean age was 41.44 years. These findings are consistent with a similar study conducted in California, where the mean age of respondents was 40.9 years. The present study found that most of the respondents were male, and this is consistent with a grounded study done in Universiti Teknologi MARA Cawangan Selangor, Malaysia, where 58% of the employees were male. However, the percentage of female employees was lower in the present study compared to the Malaysian study [2]. Before lockdown, all employees came in early, went home late, or worked on off days. After the lockdown, 94.4% of employees continued this behavior. Most of the employees had higher performance all the time before the lockdown, while only 63.3% of employees had higher performance after the lockdown. This decrease in work performance was statistically significant, and these findings are consistent with a case study conducted on jute industry workers in Kolkata [27, 29] and a grounded study done in Universiti Teknologi MARA Cawangan Selangor, Malaysia [2]. Before lockdown, most employees had very good quantity of work, and after the lockdown, 87.2% of employees had very good quantity of work. Most of the employees never had quantity of work below their expectation before and after lockdown. However, the present study did not find any published studies that compared quantity of work in factory workers pre and post lockdown. The present study also found that before lockdown, most of the employees had higher work quality all the time. After lockdown, 79.1% of employees had higher work quality all the time. This decrease in work quality after the lockdown is consistent with a case study conducted on the Indian automotive industry, which found a reduction in the quality of work due to the lockdown. Before lockdown, most of the employees never faced any problem with concentration or health issues. After lockdown, 97.4% of employees never experienced the barrier of health problems at work, while 98.5% of employees never faced concentration problems. The present study did not find any published studies that compared concentration or health issues in factory workers pre and post lockdown.

The results also showed that the work efficiency of the factory workers varied depending on the nature of the work. Workers who were involved in manual work, such as assembly line workers, were more severely affected by the lockdown than workers involved in administrative work. This can be attributed to the fact that manual work requires a physical presence at the workplace, which was not possible during the lockdown. The study found that after the lockdown was lifted, the work efficiency of the factory workers gradually improved. The workers were able to return to their normal work routine, and the challenges faced during the lockdown were gradually addressed. The study also found that the workers' work efficiency was affected by their overall health and well-being. Workers who were physically and mentally healthy were able to work more efficiently than those who were not. The findings of this study have significant implications for policymakers, employers, and employees. Policymakers need to develop strategies that support workers during periods of disruption, such as providing support for remote working and ensuring the provision of proper infrastructure and equipment. Employers need to recognize the impact of disruptions on the work efficiency of their employees and develop strategies that support their employees' well-being. Employees need to develop strategies to maintain their physical and mental well-being during periods of disruption.

The present study has several limitations that should be taken into account. Firstly, it was conducted in only one factory due to limited time and resources available, therefore, the results cannot be generalized. Secondly, the study did not consider the behavioral changes of workers during the lockdown phase. However, based on the findings of this study, some recommendations can be made. Large population-based studies should be planned to further understand the impact of the COVID-19 pandemic on the work efficiency of factory workers. Additionally, further studies need to be conducted to determine the cause behind the difference in work efficiency of factory workers before and after lockdown.

In conclusion, the COVID-19 pandemic has had a significant negative impact on the global economy and workforce, with factory workers being severely affected by pandemic-induced lockdowns. The present study aimed to investigate the impact of lockdown on the work efficiency of factory workers. The study found that the work efficiency of factory workers was significantly affected by the lockdown, with a statistically significant decrease in work performance and quality after the implementation of the lockdown. The study also provided unique insights into the socio-demographic details of factory workers. However, more research is needed to investigate the impact of lockdown on the quantity of work, concentration, and health issues among factory workers. These findings may be useful for policymakers and businesses to develop strategies to mitigate the impact of lockdown on work efficiency and ensure the well-being of their employees. Moreover, the nature of work and the lack of social interaction and physical activity have contributed to the decline in work productivity and mental health issues among factory workers during lockdowns. Thus, implementing strategies such as mental health support, physical activity promotion, and flexible work arrangements can help mitigate these negative effects and improve the work efficiency and well-being of factory workers during lockdowns. This study highlights the significant impact of the COVID-19 pandemic and subsequent lockdowns on the work efficiency of factory workers. The findings of this study provide valuable insights for policymakers, employers, and employees to develop strategies that support workers during periods of disruption.

## Conclusion

Based on the findings of the study, it can be concluded that the COVID-19 outbreak had a significant impact on the work efficiency of factory workers. The study revealed that there was a slight decrease in work efficiency after the lockdown, as evidenced by a reduction in the percentage of employees who arrived early, stayed late or worked on off days, and a decrease in performance, quantity, and quality of work. The study also found that employees experienced greater stress due to the change in work settings brought about by the pandemic. This is reflected in the reduction of employees who strongly disagreed to the statement that the stress of work due to health problems was much harder to handle after lockdown. The findings of this study provide important insights into the impact of the COVID-19 pandemic on the work efficiency of factory workers. These insights can be used to inform future policies and strategies aimed at improving the work conditions of employees during such crises. In conclusion, the study highlights the need for employers to take into account the unique challenges faced by employees during pandemics and to implement measures to support their well-being and maintain their work efficiency.

## Data Availability

The datasets generated during and/or analysed during the current study are available from the corresponding author on reasonable request.
